# Non-linear feeding functional responses in the Greater Flamingo (*Phoenicopterus roseus*) predict immediate negative impact of wetland degradation on this flagship species

**DOI:** 10.1002/ece3.554

**Published:** 2013-04-12

**Authors:** Anne-Sophie Deville, David Grémillet, Michel Gauthier-Clerc, Matthieu Guillemain, Friederike Von Houwald, Bruno Gardelli, Arnaud Béchet

**Affiliations:** 1 Centre de recherche de la Tour du ValatLe Sambuc, 13200, Arles, France; 2 Centre d'Ecologie Fonctionnelle et Evolutive, CNRS UMR 51751919 route de Mende, 34293, Montpellier cedex 5, France; 3 FitzPatrick Institute, DST/NRF Centre of Excellence, University of Cape TownRondebosch, 7701, South Africa; 4 Departement Chrono-Environnement, UMR UFC/CNRS 6249 USC INRA, Université de Franche-ComtéBesançon, France; 5 Office National de la Chasse et de la Faune Sauvage, CNERA Avifaune Migratrice, La Tour du ValatLe Sambuc, F-13200, Arles, France; 6 Basel zooBachlettenstrasse 75, 4054, Basel, Switzerland

**Keywords:** *Artemia spp*, attack rate, conservation, filter feeder, food intake rate, handling time, salt pans

## Abstract

Accurate knowledge of the functional response of predators to prey density is essential for understanding food web dynamics, to parameterize mechanistic models of animal responses to environmental change, and for designing appropriate conservation measures. Greater flamingos (*Phoenicopterus roseus*), a flagship species of Mediterranean wetlands, primarily feed on *Artemias* (*Artemia spp*.) in commercial salt pans, an industry which may collapse for economic reasons. Flamingos also feed on alternative prey such as Chironomid larvae (e.g., *Chironomid spp*.) and rice seeds (*Oryza sativa*). However, the profitability of these food items for flamingos remains unknown. We determined the functional responses of flamingos feeding on *Artemias,* Chironomids, or rice. Experiments were conducted on 11 captive flamingos. For each food item, we offered different ranges of food densities, up to 13 times natural abundance. Video footage allowed estimating intake rates. Contrary to theoretical predictions for filter feeders, intake rates did not increase linearly with increasing food density (type I). Intake rates rather increased asymptotically with increasing food density (type II) or followed a sigmoid shape (type III). Hence, flamingos were not able to ingest food in direct proportion to their abundance, possibly because of unique bill structure resulting in limited filtering capabilities. Overall, flamingos foraged more efficiently on *Artemias*. When feeding on Chironomids, birds had lower instantaneous rates of food discovery and required more time to extract food from the sediment and ingest it, than when filtering *Artemias* from the water column. However, feeding on rice was energetically more profitable for flamingos than feeding on *Artemias* or Chironomids, explaining their attraction for rice fields. Crucially, we found that food densities required for flamingos to reach asymptotic intake rates are rarely met under natural conditions. This allows us to predict an immediate negative effect of any decrease in prey density upon flamingo foraging performance.

## Introduction

Global environmental change affects the whole biosphere, from individual organisms, species, and ecosystems to entire biogeochemical cycles (Vitousek et al. 1997; Milly et al. 2005). Great emphasis has been given to the biodiversity crisis and the ongoing ‘sixth extinction’ (e.g., Thomas et al. 2004), yet one of the main impacts of global change is to deeply modify biological interactions (Petchey et al. 1999; Eisenhauer et al. 2012). It is therefore essential to study species relationships in a changing world, in particular trophic relationships that condition nutrient flux and shape food webs (Pimm 1982; Williams and Martinez 2000).

Functional relationships quantifying changes in predators intake rate relative to prey density (Solomon 1949) are key elements for understanding habitat selection, food resource preferences (Mysterud and Ims 1998), food webs, and hence general predator–prey interactions (Dale et al. 1994; Barnhisel and Kerfoot 2004). Functional response measurements provide two parameters: the *attack rate* (instantaneous rate of food discovery) and the *handling time* (time required to extract and ingest food; Holling 1959). The value of these parameters can differ according to food item (Badii et al. 2004) and/or substrate types (Kuhlmann and Hines 2005). Functional responses typically inform about (1) the foraging effort necessary to balance the energy budget of a predator feeding on a given resource, (2) the threshold prey density below which sustainable foraging is compromised (Enstipp et al. 2007), and (3) foraging efficiency depending on prey type.

Accurate knowledge of functional relationships in a changing world is therefore essential for the management of threatened species (Whittingham and Markland 2002; Grémillet and Charmantier 2010). Specifically, studying functional responses can help focusing conservation efforts on the predator's most profitable prey (Rubega and Inouye 1994). Identifying and quantifying functional responses are also essential for the design of mechanistic models, which are being increasingly used to predict the responses of animal populations to environmental change (Pettifor et al. 2000; Kearney and Porter 2009; Kearney et al. 2009; Stillman and Goss-Custard 2010).

Holling's theory (Holling 1959) describes three main types of functional responses. Type I corresponds to a linear increase in intake rate with increasing food density, up to a threshold level beyond which it remains constant. Type II shows an increasing intake rate with an asymptotic form, and type III presents a sigmoid shape. According to theory, type I response is exclusive to filter feeders (Holling 1965) as they are theoretically not limited by food processing. This is explained by their ability to capture several food items simultaneously and the relative small size and immobility of their food compared with those of nonfilter feeders (Jeschke et al. 2004).

Empirical assessments of the three types of functional responses have been subjected to detailed investigations in invertebrates (see Jeschke et al. 2004; for a review). However, for filter-feeding invertebrates, several empirical results do not support the prediction of a linear relationship between food density and food intake rate (reviewed in Jeschke et al. 2004). For instance, a type III functional response was found in *Daphnia magna* (Cladocera: Crustacea, Porter et al. 1983), in limnetic suspension feeders (Chowfraser and Sprules 1992) and in the burrowing shrimp (*Upogebia deltaura*, Lindahl and Baden 1997). However, functional responses are often difficult to assess in vertebrates filter feeders for logistic reasons, especially in large species. Such problems have, however, been overcome in a limited number of such species, yielding to definition of functional responses for fish (Ivlev 1961; Houde and Schekter 1980; Durbin and Durbin 1981; Miller et al. 1992; Lynch 2007), Common teal (*Anas crecca*; Arzel et al. 2007), or Minky whale (*Balaenoptera acutorostrata*; Smout and Lindstrom (2007). Results concluded to a type I functional response for Teal, as predicted by theory. Conversely, depending on the species a type II or III best fitted the data for fish, and a type III was found for the Minky whale.

Here, we investigated the functional relationships in the greater flamingos (*Phoenicopterus roseus*, hereafter, ‘flamingos’), a flagship bird species of Mediterranean wetlands. Flamingos are filter feeders (Jenkin 1957) using their unique bill structure to harvest a diversified diet including aquatic invertebrates and seeds (Johnson and Cézilly 2007).

In the Mediterranean, five of the nine major flamingo breeding sites are located in commercial salt pans (Johnson and Cézilly 2007). In these areas, the artificial and predictable impoundment with a high salt concentration allows the development of high densities of brine shrimps in the water column (*Artemia*s *spp*.). Brine shrimps are the main prey of flamingos during the breeding period (Britton and Johnson 1987; Béchet and Johnson 2008). Nevertheless, Chironomid larvae (e.g., *Chironomus* spp., *Cricotopus* spp., *Paratanytarsus grimii, Tanytarsus volgensis*, *Halocladius varians*) are important alternative prey that flamingos can find in the sediment of most ponds (Britton et al. 1986; Johnson and Cézilly 2007). In the Camargue (Southern France), salt production has recently ceased over half of the surface area (6000 ha) of what was the largest commercial salt pan in Europe, Salin-de-Giraud. The activity over the remaining production area (5000 ha) might also cease in the near future (Béchet et al. 2009, 2012). This discontinuation of artificial impoundment and upheavals of the physicochemical conditions at the origin of the high concentrations of *Artemias* may accelerate food depletion and increase intraspecific competition by higher densities of flamingos in the alternative habitat types (Sutherland and Anderson 1993; Béchet and Johnson 2008). Alternative habitats include freshwater marshes and natural brackish lagoons. In spring, flamingos can also forage in freshly sown rice fields causing important crop damage (Fasola and Ruiz 1996; Tourenq et al. 2001). The use of these agricultural areas by flamingos could increase with increasing decline in natural wetlands (Czech and Parsons 2002).

Our objective is to evaluate Holling's predictions in flamingos, a vertebrate filter feeder, for different food items in order to better assess how habitat changes might affect its foraging performance, and hence, its population dynamics across the Mediterranean. We predicted that flamingos should show a type I functional response with possible different *attack rates* and *handling times* between prey types. We experimentally determined the functional responses of flamingos to varying densities of three prey types: (1) *Artemia,* (2) Chironomid larvae, and (3) rice (*Oryza sativa*).

## Material and Methods

### Experimental design

Experiments were carried out on captive flamingos at Basel zoo (Switzerland). A first experimental session took place in February and March 2011 and a second one in November 2011. For each session, 11 adult birds (six males and five females) were randomly selected from a flock of 112 individually ringed flamingos. We therefore used a total of 22 birds for the whole experiment (11 for the first session and 11 for the second one). Birds were kept in an outdoor exhibit, and moved to a 15 m² indoor experimental aviary. The aviary ground was covered with soft flooring adapted to flamingo feet and a pond of 3 m² was available at one end of the aviary. Most birds were born in captivity and therefore used to human presence. The birds were moved to the indoor aviary 1 week before the experiments to habituate to this new environment. The study was approved by the ethics committee of Basel zoo, and birds were monitored by veterinarians all along the experiment.

We successively offered different prey densities to flamingo foraging in a 28 × 28 cm² tray (13.5 cm depth) positioned in front of the pond. This setup allowed flamingos to filter feed without spilling food outside the tray with their feet. To feed, flamingos draw water through the tip of their bills with their tongue in a rapid back and forth piston-like movement creating suction. The water crosses the platelets of lamellae, which retain the food, and is expelled near the base of the bill (Jenkin 1957).

Because flamingos are colonial birds and need to be in group to forage, it was not possible to test them individually during functional response trials. We therefore used two other trays to distribute the birds as we aimed at obtaining individual measures of intake rate on the experimental tray. These additional trays were filled with food pellets. Birds were food deprived 12 h before each session. Sessions of four subsequent trials performed at 1-hour interval were conducted. As flamingos were given very few prey items at each trial and ate very few pellets, we expected their foraging effort to remain constant across all four trials. Flamingos subsequently complemented their meals with food pellets. A video camera allowed recording the birds' behavior from a hide, including their feeding time (bill underwater), without disturbing them. Trials never lasted more than a few minutes in order to avoid food depletion, as recommended by Royama (1971) and Fritz et al. (2001). The order in which the different food densities were offered was randomized. Intake rates were calculated as the amount of food item consumed (difference in the number of food items counted in the tray before and after each trial) divided by individual feeding time (Pettifor et al. 2000; Arzel et al. 2007). But although we placed three trays to get only one bird per trial, in 49% of the cases for *Artemias*, 79% for Chironomid larvae, and 71% for rice, up to four individuals were observed eating simultaneously in the experimental tray. When more than one bird fed in the tray during a trial, it was therefore considered that all individuals had ingested the same amount of food per second, that is, their intake rate was the same. Intake rates were thus calculated as the number of food items consumed, divided by ‘collective feeding times’, corresponding to the sum of time spent bill underwater by the *n* birds observed feeding in the tray.

In some cases birds could switch between the feeding trays during an experiment (from pellets to the experimental tray). However, as intake rate is influenced by beak features and current food characteristics (size, consistency, and substrate) rather than the characteristics of previously ingested food items, and as birds could not feed to satiety during the experiments (each experimental trial was limited in time, and birds generally started to feed on their usual food at the end of the morning experiments), we are confident that this did not significantly affect the estimation of food intake rates.

#### Food items

*Artemias* – We used a mix of juvenile and adult *Artemias* (*Artemia spp*.) sampled from the Camargue wetlands (Southern France) and kept them alive in a tank containing phytoplankton. The time between *Artemias* sampling and their use for trials was 1–4 days. *Artemias* were on average 8.21 mm (±2.80) long and 2.50 mm (±1.22) width (*n* = 50, all measures on food items' size are given ±SE). We first determined the number of *Artemias* per gram by counting the number of individuals on photographs of 1, 2.5, 5, 15, and 50 g of fresh individuals spread over a gridded tray. As the relationship between the weight and the number of *Artemias* was linear (*R*² = 0.83, *P* < 0.001), we used *Artemia* fresh weight as a proxy for food quantity. We then presented amounts of 1, 2.5, 5, 15, 30, 50, 90, 150, and 200 g of *Artemias* per tray, corresponding to a range of 130–26,000 individuals. For each trial, *Artemias* were placed in 6 liters of sea water so that the maximum density tested was 13 times the maximum density encountered in the wild (Britton and Johnson 1987). Four to six trials were performed per density, resulting in 43 separate *Artemia* trials over 3 weeks.

Chironomid larvae – We used alive, commercially available, freshwater Chironomid larvae. Their size (0.94 cm in length ±0.20; and 0.080 cm in width ±0.012; *n* = 50) was within the range of sizes of species found in Camargue salt pans (0.84 cm in length ±0.13; and 0.091 cm in width ±0.014 cm, *n* = 50) and freshwater ponds (1.86 cm in length ±0.49; and 0.119 cm in width ±0.0311 cm, *n* = 50). In natural conditions, Chironomid larvae burrow in the first centimeters of the sediment (Britton and Johnson 1987; Johnson and Cézilly 2007). Hence, for each trial, larvae were placed between two layers of sand grains, each layer measuring 2 cm (diameter 0.1–1 mm), and four liters of freshwater were added to mimic natural conditions. Water was therefore turbid and flamingos could not detect prey visually. Larvae numbers were individually counted using a sieve to separate Chironomid larvae from the sediment, before and after each trial. We offered 5, 10, 20, 30, 50, 70, 100, 150, 200, or 300 Chironomid larvae per trial, with five replicates per density. Because intake rate was still increasing between 200 and 300 larvae, we added three replicates at 600 individuals in order to search for a possible asymptotic intake rate. Six hundred larvae is 12 times the maximum density that can be encountered in salt pans (Britton and Johnson 1987). The dataset resulted in 53 separate trials over a period of 3 weeks.

Rice – We used one of the most common rice varieties of the Camargue (“Arelate”, long rice seed). Seeds were 0.97 ± 0.039 cm long and 0.26 ± 0.024 cm width (*n* = 50). During planting by rice farmers, grains are simply laid on the sediment before flooding, allowing germination. Hence, to mimic natural conditions, seeds were laid over a 4 cm layer of sand (diameter 0.1 to 1 mm) and covered with 4 liters of fresh water, to reach an 8 cm water depth and mimic natural rice field conditions. We offered 50, 100, 300, 600, 1000, 2000, 3000, or 4000 rice seeds per tray, representing a range between 0.5 kg and 40 kg m^−2^ of dry rice. As the intake rate was still increasing from 3000 to 4000 rice seeds, we added three trials with 6000 seeds to search for a possible asymptotic intake rate. This maximum seed density was approximately 80 times the density sown in rice fields, and 10 times that encountered in hunting marshes where owners seed-bait to attract game (A.-S. Deville, pers. data). Seeds were counted before and after each trial. Six trials were performed for each density (except the highest one for which there were only three trials), resulting in 51 separate trials over a period of 3 weeks.

### Modeling functional responses

The two main parameters affecting the shape of the functional response are as follows: (1) the *attack rate* (*a*), representing the rate at which a predator encounters its prey; and (2) the *handling time* (*h*), representing the time needed for capturing and ingesting a food item (Holling 1959). In our study, *a* was the mean instantaneous quantity of prey encountered during the entire foraging trial (expressed as the number of prey or seeds per unit of time), and *h* was the time needed for handling prey or seeds in the water and/or in the mud, process it with the bill, and ingest it. A predator with negligible handling time keeps up with increasing prey densities by eating them in direct proportion to their abundance in the environment. Its intake rate therefore increases linearly with increasing food density. Nevertheless, this linear increase ceases at a maximum food density beyond which the intake rate becomes constant (Begon et al. 1990). This type I functional response is defined by the following equations:


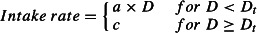
(1)

where *D* is prey density and *D*_*t*_ is the threshold density beyond which intake rate remains constant and equal to *c*.

When consumers require a non-negligible handling time to ingest their prey, the intake rate initially rises quickly as the density of prey increases, but then decelerates asymptotically toward a plateau. Such consumers present a type II functional response, which is the most commonly found response (Jeschke et al. 2004). A type II response becomes a type III if consumers require learning or switch between food types, patches, or foraging tactics (Jeschke et al. 2004). Their intake rate therefore remains low at low prey densities. Types II and III should follow equation:


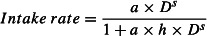
(2)

If *s* = 1, the curve is of type II (also called Holling's Disk Equation) while values of *s* > 1 correspond to a type III sigmoid shape.

### Statistical analyses

We evaluated the fit of the three types of functional responses, both within the whole range of food item densities considered in our experiments (hereafter ‘experimental range’) and within natural density ranges (hereafter ‘natural range’). To assess whether the number of flamingos feeding in the tray affected the intake rate measurements, we evaluated the fit of the three types of functional responses separately for data obtained on single individuals and for those resulting from groups of individuals feeding together. If the same functional response type was found in both cases, data from both sources were pooled to maximize statistical power. This procedure was repeated for each food type.

We used linear and nonlinear models to assess variations in intake rates with increasing resource densities. Type I functional responses were assessed by fitting a linear relationship between intake rate and food density using equation (1). Type II and type III functional responses were fitted with package ‘nlme’ in R (R Development Core Team 2012) using equation (2). We forced *s* = 1 to evaluate the type II response and different values of *s* > 1 to evaluate the fit of type III responses. Values of *s* between 1.01 and 5 were used following Smout and Lindstrom (2007), who fitted functional responses for filter-feeding Minky whales (*Balaenoptera acutorostrata*). Additionally, to evaluate if relationships could be considered as the first term of an expansion of a type III response (first part of the curve), we also tested for an exponential function (hereafter ‘Partial type III’): 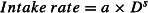 (Gentleman et al. 2003; Morozov 2010).

Attack rate *a* and handling time *h* were inferred from the best model rather than being recorded directly during the experiments, which was impossible as most bill action occurs under water. Models were selected based on the Akaike Information Criterion with adjustment for small sample sizes (AICc, Burnham and Anderson 2002). *s* was considered as an extra parameter when calculating the AICc for both types III models. According to the equations of types II and III models, intake rate is equal to zero at food density = 0. In contrast, type I functional response with nonzero intercept is occasionally found in the literature (da Rocha and Redaelli 2004; Durant et al. 2009). Hence, for types I functional response we evaluated models where the intercept of intake rate was forced to zero or not when food density was equal to zero. Finally, when the AICc method did not allow discriminating between several mechanistic models of functional responses (ΔAICc < 2), we used a logistic regression of the consumption rate of food items as a function of the log-transformed food density (Trexler et al., 1988). Examining the first term of a cubic regression allows diagnosing the shape of the density-dependent rate of food consumption. A null linear parameter indicates a type I functional response, a significant negative linear parameter indicates a type II, whereas a positive linear parameter indicates a type III response (Trexler et al., 1988).

## Results

### Artemias

**Figure 1:**
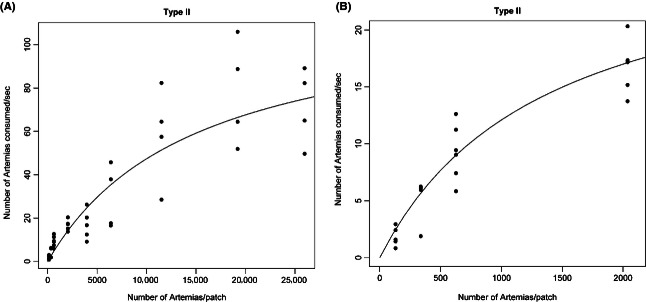
Intake rate (number of *Artemias* consumed per second) of flamingos as a function of *Artemias* density (number of *Artemias* per patch). (A) Densities from 130 to 26,000 *Artemias* per tray (i.e., for the experimental range) for the best model. (B) Densities from 130 to 1950 *Artemias* per tray (i.e., for the natural range) for the best model 169 × 169 mm (300 × 300 DPI).

Only five different birds fed during the *Artemia* experimental trials. Intake rates were thus computed for this number of individuals only. As we found the same functional response type when using only data from single individuals or groups of birds (ΔAICc < 2), data from both sources were pooled. Type II functional response was retained both for the experimental and the natural range of prey densities (ΔAICc < 2, Table 1A and B). We calculated the asymptotic intake rate corresponding to ratio 1/*h*. Within the experimental range of prey densities (Fig. 1A), the plateau was 118 *Artemias* sec^−1^ and 29 *Artemias* sec^−1^ for the natural range (Fig. 1B).

**Table 1:** Model selection for types I, II, and III functional responses of flamingo feeding on *Artemias*: (A) for the experimental range of densities (from 130 to 26,000 *Artemias* per tray) and (B) only for the natural range of densities (from 130 to 1950 *Artemias* per tray)

Holling model type	Intake rate	K	Deviance	AICc	Δ AICc	AICc weights (%)	Parameter estimates
(A)							
**Type II**	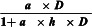	**3**	**−170.90**	**348.42**	**0**	**74.80**	***a*** **= 0.0079 (±0.0016)** ***P*** **< 0.001** ***h*** **= 0.0085 (±0.0016)** ***P*** **< 0.001**
Full Type III	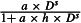	4	−170.79	350.63	2.21	24.77	
Type I	*a* × *D* + *β*	3	−176.15	358.91	10.49	0.39	
Type I (through zero)	*a* × *D*	2	−180.11	364.53	16.11	0.024	
Partial Type III	*a* × *D*^*s*^	3	−180.36	367.34	18.92	0.0058	
(B)							
**Type II**	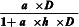	**3**	**−43.28**	**93.97**	**0**	**75.18**	***a*** **= 0.021 (±0.0030)** ***P*** **< 0.001** ***h*** **= 0.035 (±0.0053)** ***P*** **< 0.001**
Full Type III	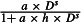	4	−42.88	96.26	2.29	23.93	
Type I	*a* × *D* + *β*	3	−47.74	102.89	8.92	0.87	
Type I through zero	*a* × *D*	2	−53.00	110.66	16.69	0.018	
Partial Type III	*a* × *D*^*s*^	3	−53.23	113.87	19.90	0.0036	

For the type III functional response, both the entire shape (‘Full Type III’) and the first exponential part of a sigmoid (‘Partial Type III’) were tested. Only results with the value of s (‘shape parameter’) giving the best AICc are presented. The best models are indicated in bold. K corresponds to the number of parameters, IR designs intake rate, D is the food density, s the ‘shape parameter’, *a* the attack rate (in number of *Artemias*/second), and *h* the handling time (in sec). β is the intercept of a type I not forced through zero. Parameter estimation (±SE) is given for the best model(s) only.

### Chironomid larvae

**Figure 2:**
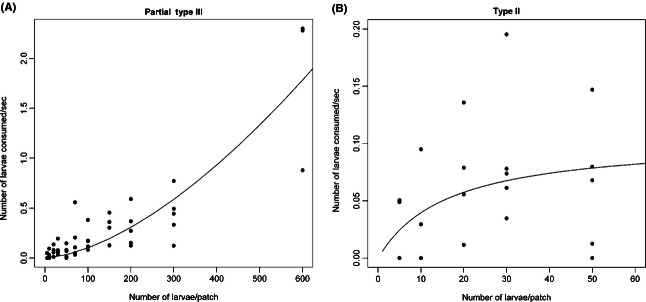
Intake rate (number of Chironomid larvae consumed per second) of flamingos as a function of Chironomid density (number of Chironomid larvae per patch). (A) Densities from 5 to 600 larvae per tray (i.e., for the experimental range) for the best model. (B) Densities from 5 to 50 larvae per tray (i.e., for the natural range) for the best model 169 × 169 mm (300 × 300 DPI).

Eight different birds were fed during the Chironomid larvae trials. As we found the same functional response type when using only data from single individuals or from groups of birds (ΔAICc < 2), data from both sources were pooled. In flamingos feeding on Chironomid larvae within the experimental range of prey densities, our analysis identified both types II and III as the possible best model (ΔAICc > 2, Table 2A). However, none of these two models was biologically acceptable because handling time was found to be negative. As this is likely a mathematical artifact which has no biological rationale, we followed Song and Heong (1997), Sing and Arbogast (2008), and Kratina et al. (2009) and retained the next model, corresponding to the Partial type III (Table 2A and Fig. 2A). In contrast, within the natural range of prey densities, we could not discriminate between type II, full type III, and type I responses (ΔAICc < 2, Table 2B and Fig. 2B). The linear term of the logistic regression of prey consumption rate was negative (Table 3) indicating a type II response. Flamingos thus showed a type II response when feeding on chironomid larvae within the natural range of densities. The plateau within this range corresponded to 0.104 larvae sec^−1^.

**Table 2:** Model selection for types I, II, and III functional responses of flamingo feeding on Chironomid larvae: (A) for the experimental range of densities (from 5 to 600 Chironomid larvae per tray) and (B) only for the natural range of densities (from 5 to 60 Chironomid larvae per tray)

Holling model type	Intake rate	K	Deviance	AICc	Δ AICc	AICc weights (%)	Parameter estimates
(A)							
Type II	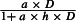	3	8.35	−10.21	0	70.69	*a* = 0.0012 (±0.00023) *P* < 0.001 *h* = −0.082 (±0.00014) *P* < 0.001
Full Type III	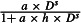	4	8.32	−7.79	2.42	21.08	*a* = 0.0012 (±0.00022) *P* < 0.001 *h* = −0.80 (±0.26) *P* < 0.001
**Partial Type III**	***a*** **×** ***D***^***s***^ **(*****s*** **= 1.60)**	**3**	**6.20**	**−5.90**	**4.31**	**8.19**	***a*** **= 0.000064 (±0.000004006)** ***P*** **< 0.001**
Type I through zero	*a* × *D*	2	−0.85	5.95	16.16	0.022	
Type I	*a* × *D* + *β*	3	0.030	6.44	16.65	0.017	
(B)							
**Type II**	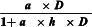	**3**	**39.43**	**−71.65**	**0**	**42.23**	***a*** **= 0.0064 (±0.0052)** ***P*** **= 0.23** ***h*** **= 9.59 (±4.76)** ***P*** **= 0.056**
**Type I through zero**	***a*** **×** ***D***	**2**	**37.34**	**−70.11**	**1.54**	**19.55**	***a*** **= 0.0019 (±0.00038)** ***P*** **< 0.001**
**Type I**	***a*** **×** ***D*** **+** ***β***	**3**	**38.52**	**−69.85**	**1.80**	**17.17**	***a*** **= 0.0011 (±0.00064)** ***P*** **= 0.099**
**Full Type III**	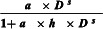 **(*****s*** **= 3.2)**	**4**	**39.91**	**−69.71**	**1.94**	**16.01**	***a*** **= 0.0062 (±0.0051)** ***P*** **= 0.23** ***h*** **= 9.65 (±4.72)** ***P*** **= 0.052**
Partial Type III	*a* × *D*^*s*^	3	36.91	−67.40	4.25	5.04	

For the type III functional response, both the entire shape (‘Full Type III’) and the first exponential part of a sigmoid (‘Partial Type III’) were tested. Only results with the value of s (‘shape parameter’) giving the best AICc are presented. The best model is indicated in bold. K corresponds to the number of parameters, IR designs intake rate, D is the food density, s the ‘shape parameter’, *a* the attack rate (in number of Chironomid larvae/second), and *h* the handling time (in sec). β is the intercept of a type I not forced through zero. Parameter estimation (±SE) is given for the best model(s) only.

**Table 3:** Maximum likelihood estimate from logistic regression of proportion of Chironomid larvae eaten as a function of initial larvae density by flamingos for the natural range of densities

Parameter	Estimate (±SE)	*t*-value	*P*
Constant	−0.93 (±0.10)	−9.17	<0.001
**Linear**	**−2.02 (±0.55)**	**−3.65**	**<0.001**
Quadratic	−0.54 (±0.50)	−1.07	0.28
Cubic	−0.43 (±0.48)	−0.90	0.37

The linear parameter is indicated in bold.

### Rice seeds

**Figure 3:**
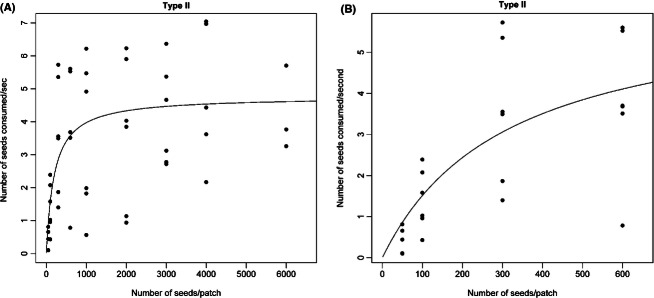
Intake rate (number of rice seeds consumed per second) of flamingos as a function of rice seeds density (number of rice seeds per patch). (A) Densities from 50 to 6000 rice seeds per tray (i.e., for the experimental range) for the best model 169 × 169 mm (300 × 300 DPI). (B) Densities from 50 to 600 rice seeds per tray (i.e., for the natural range) for the best model 169 × 169 mm (300 × 300 DPI).

Ten different birds fed during the rice experimental trials. If we considered data obtained for single individuals, types I, II, and III responses were retained (*n* = 14; ΔAICc < 2), whereas considering intake rates from more than one bird (*n* = 36), only types II and III were retained as the best responses (ΔAICc < 2). As a linear type I was not found neither for *Artemias* nor for Chironomid larvae, it is unlikely that a type I was the best response for rice, especially as the size and the consistency of this food item are higher compared with the two other food tested. As the difficulty to discriminate between the three models is likely caused by small sample size from trials with single individuals, we decided to pool data from both sources. For flamingos feeding on rice, we could not distinguish the best response between type II and full type III, both for the experimental density range and for the natural density range (ΔAICc < 2, Table 4A and B; Fig. 3A and B). The logistic regression indicated a significant negative linear parameter for both ranges, suggesting a type II functional response (Table 5). Asymptotic intake rate was 5 seeds sec^−1^ within the experimental density range and 6 for naturally occurring densities.

**Table 4:** Model selection for types I, II, and III functional responses of flamingo feeding on rice seeds: (A) for the experimental range of densities (from 50 to 6000 rice seeds per tray) and (B) only for the natural range of densities (from 50 to 600 rice seeds per tray)

Holling model type	Intake rate	K	Deviance	AICc	Δ AICc	AICc weights (%)	Parameter estimates
(A)							
**Type II**	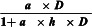	**3**	**−96.45**	**199.42**	**0**	**67.52**	***a = 0.024 (±0.0099) P = 0.019*** ***h = 0.21 (±0.020) P < 0.001***
**Full Type III**	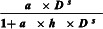 **(*****s*** **= 1.8)**	**4**	**−96.00**	**200.89**	**1.47**	**32.38**	***a = 0.00053 (±0.00029) P = 0.08*** ***h = 0.24 (±0.018) P < 0.001***
Type I	*a* × *D* + *β*	3	−102.97	212.46	13.04	0.10	
Type I through zero	*a* × *D*	2	−117.04	238.33	38.91	0	
Partial Type III	*a* × *D*^*s*^	3	−117.22	240.96	41.54	0	
(B)							
**Type II**	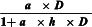	**3**	**−39.49**	**86.19**	**0**	**50.45**	***a*** **= 0.020 (±0.0082)** ***P*** **= 0.024** ***h*** **= 0.16 (±0.054)** ***P*** **= 0.0062**
**Full Type III**	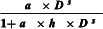 **(s = 2)**	**4**	**−38.40**	**86.90**	**0.71**	**35.38**	***a*** **= 0.00022 (±0.00011)** ***P*** **= 0.055** ***h*** **= 0.24 (±0.031)** ***P*** **< 0.001**
Type I through zero	*a* × *D*	2	−42.48	90.34	4.15	6.33	
Type I	*a* × *D* + *β*	3	−41.58	90.36	4.17	6.27	
Partial Type III	*a* × *D*^*s*^	3	−42.97	93.14	6.95	1.56	

For the type III functional response, both the entire shape (‘Full Type III’) and the first exponential part of a sigmoid (‘Partial Type III’) were tested. Only results with the value of s (‘shape parameter’) giving the best AICc are presented. The best model is indicated in bold. K corresponds to the number of parameters, IR designs intake rate, D is the food density, s the ‘shape parameter’, *a* the attack rate (in number of seeds/sec), and *h* the handling time (in sec). β is the intercept of a type I not forced through zero. Parameter estimation (±SE) is given for the best model(s) only.

**Table 5:** Maximum likelihood estimate from logistic regression of proportion of rice seeds eaten as a function of initial seeds density by flamingos: (A) for the experimental range of densities and (B) for the natural range of densities

Parameter	Estimate (±SE)	*t*-value	*P*
(A)			
Constant	−2.13 (±0.11)	−20.14	<0.001
**Linear**	**−6.04 (±1.03)**	**−5.84**	**<0.001**
Quadratic	−2.90 (±0.83)	−3.52	<0.001
Cubic	1.01 (±0.64)	1.59	0.12
(B)			
Constant	−2.68 (±0.11)	−23.63	<0.001
**Linear**	**−6.04 (±0.59)**	**−10.25**	**<0.001**
Quadratic	−0.83 (±0.56)	−1.50	0.15
Cubic	0.34 (±0.52)	0.66	0.52

The linear parameter is indicated in bold.

## Discussion

On the basis of our findings, we reject the theoretical prediction of a linear type I shape for the functional response of Greater flamingos feeding on a set of various prey types. Crucially, we show that the intake rate always varies nonlinearly with increasing food densities, and that both *attack rate* and *handling time* depend on prey types (Table 6 and Figs 3). Nonlinear functional responses suggest that, contrary to theoretical predictions for filter feeders (Holling 1965), flamingos were not able to ingest food items in direct proportion to their abundance. This rather surprising result might be due to the structural limit set upon intake rates by the maximum volume of water which can be filtered through the flamingo's bill per unit of time, or a possible clogging of the lamellae (Guillemain et al. 1999).

**Table 6:** Overall summary of results for functional responses of flamingos feeding on *Artemias*, Chironomid larvae, and rice seeds

Food item	Range	Type of functional response retained	Asymptotic intake rate
*Artemias*	Experimental	Type II	118 prey/sec
	Natural	Type II	29 prey/sec
Chironomid larvae	Experimental	First part of a type III	
	Natural	Type II	0.104 prey/sec
Rice seeds	Experimental	Type II	5 seeds/sec
	Natural	Type II	6 seeds/sec

Experimental and natural ranges of food densities are presented with the corresponded functional response and asymptotic intake rate (i.e., the maximum intake rate).

Our results could be affected by the fact that we used captive birds for which aquatic invertebrates or rice seeds were not the food provided by the zoo personal on a daily basis. However, these birds are often seen foraging on invertebrates in the artificial pond of their enclosure, so invertebrates were not a new food type to them. It is also unlikely that regular feeding on pellets (i.e., captivity conditions) has an impact on bill morphology (lamellae) and so on intake rates, as selection pressure does not operate within so few generations in captivity (Champagnon et al. 2010). In our case, birds were from the F0 to the F6 generation. Performing such feeding experiments with wild-caught individuals would in any case be both ethically and scientifically ineligible because of the stress level precluding normal behavioral patterns in wild birds once captured. Furthermore, attracting wild flamingos to feed in an artificial pond with known densities of single prey types seems far from realistic as this species is notoriously shy in the wild. There is consequently no current alternative to our experimental setup, and our study does provide essential information about the functional responses of a vertebrate filter feeder, as well as major insights into flamingo foraging ecology.

Jeschke et al. (2004), who investigated such processes in invertebrates, suggested that a consumer must fulfill two conditions to show a type I functional response: (1) its handling time must be negligible (‘handling condition’); and (2) unless its gut is completely filled and gut passage time is minimal, the consumer must search for food at a maximal rate with maximum effort (‘satiation condition’). According to previous studies, only filter feeders may meet both of these conditions (see for instance, Rigler 1961 and Wilhelm et al. 2000 for branchiopods, Frost 1972 for copepods, and Rothhaupt 1990 for rotifers). Thus, flamingos do not meet at least one of these conditions. We think that the ‘satiation condition’ is met as birds in the experimental setup were most probably never satiated. Indeed, wild flamingos have to spend approximately 40% of their total daily activity feeding on invertebrates (Britton et al. 1986; Galicia and Baldassarre 1997), while in our experiment birds only had time to feed on a limited number of prey items during each session, and subsequently complemented their meals with food pellets. Hence, it is legitimate to assume that flamingos involved in our experiment searched for food at a maximal rate with maximum effort. Conversely, flamingos did not fulfill the handling condition, as handling times recorded for the different prey types were not negligible, especially for Chironomid larvae (conversely, attack rates values often overlap zero, meaning that flamingos are very quick and efficient in encountering food items).

One of the observed functional responses was also in accordance with a partial type III (i.e., for the experimental range of Chironomids densities). According to the literature, two main mechanisms may cause a type III response. (1) It may occur when predators increase their search activity with increasing prey density (‘learning time’). For instance, many predators respond to kairomones and increase their activity levels in the presence of prey (Vanalphen and Galis 1983). (2) It may also be observed when predators such as polyphagous vertebrates switch between food types, food patches, or foraging tactics, to target the most abundant prey species once identified (Schenk and Bacher 2002). This latter option cannot be retained as it was not tested in our experimental design and because birds always filtered and never changed their foraging tactics. Here, we rather suggest that the type III response was possibly observed because flamingos increased their searching activity with increasing larvae density, or kept the same foraging effort whatever the prey density but only managed to extract Chironomids from the sediment above a certain prey density threshold.

Overall, our results suggest a reappraisal of Holling's theoretical predictions on vertebrates filter feeders (Holling 1959). Although this experiment needs to be replicated on others filter species, our results suggest that some filter feeders may be more limited than expected in their capabilities to ingest food in direct proportion to food density. Moreover, observed functional responses tend to be more variable than the three clear-cut, theoretical types, with possible intermediate responses (Williams and Martinez 2000; Okuyama 2012), which should be integrated in future research.

Beyond these theoretical considerations, our study has major implications for the conservation and management of flamingos. We compared the energetic gain per unit of time when flamingos forage on *Artemias* and on Chironomid larvae when considering the mean density of these prey in summer in salt pans (based on Britton and Johnson 1987). We found that, despite a higher energetic content in Chironomid larvae (Chironomid larvae, mean = 0.0158 kJ/larvae; Nudds and Bowlby 1984; Johnston and Cunjak 1999; and *Artemias*, mean = 0.00568 kJ/*Artemia,* Caudell and Conover 2006), flamingos have an energetic gain more than 23 times higher when feeding on *Artemias* (IR = 0.028 kJ sec^−1^) compared with Chironomid larvae (IR = 0.0012 kJ sec^−1^, calculations based on results from the natural range of prey densities). This stresses the importance of *Artemias* for flamingo populations. Additionally, this suggests that extracting prey items from the sediment is more time consuming for flamingos compared with feeding in the water column, probably because in the former case the visibility of prey is reduced and flamingos' bill lamellae can be saturated by sediment particles, slowing the handling of prey. But in the wild, flamingos often perturb the sediment with their feet (a behavior called ‘stamping-marking’; Johnson and Cézilly 2007) when feeding on benthic invertebrates such as Chironomid larvae. It is likely that under natural conditions, flamingos adopt this foraging behavior to get benthic prey to float in the water column and hence reduce handling time, particularly when prey density is low. This emphasizes the importance of considering substrate types when investigating filter-feeder foraging performance.

The energetic gain calculated for flamingos feeding on rice, when considering mean rice density in freshly sown rice fields (821 seeds m^−2^), helps in explaining their important use by flamingos (Tourenq et al. 2001). Indeed, flamingo energetic gain is 16 times higher when feeding on rice (IR = 0.45 kJ sec^−1^) compared with *Artemias*, and more than 375 times if compared with Chironomid larvae (rice, mean energetic content = 0.41 kJ/seed, calculations based on results from the natural range of prey densities). Finally, under natural conditions food densities required for flamingos to reach asymptotic intake rates are rarely met for any of the food items presented in this study (Britton and Johnson 1987). As flamingos already spend a large proportion of the day feeding (around 40%), any decrease in prey density could negatively impact their foraging payoffs.

The majority of Mediterranean flamingos forage in commercial salt pans, which harbor high invertebrate biomass, especially *Artemias*. However, >50% of such habitats have been abandoned over the last 50 years (López et al. 2010) resulting in a lower profitability for flamingos highly dependent on salt pans (Béchet and Johnson 2008) and to the development of competing land uses, such as tourism or industry (Weber et al. 1999; Masero 2003; Ortega et al. 2004). In this context, functional relationships such as those determined in our study are key input for mechanistic models required to predict individual energy budgets (Kearney and Porter 2004; Fort et al. 2009). This information can then be amplified within individual-based models, to predict population responses to potential habitat changes such as those faced by flamingos across the Mediterranean (Fargione et al. 2008).
